# Hidden Tensor Structures

**DOI:** 10.3390/e26020145

**Published:** 2024-02-07

**Authors:** Marek Czachor

**Affiliations:** Instytut Fizyki i Informatyki Stosowanej, Politechnika Gdańska, 80-233 Gdańsk, Poland; mczachor@pg.edu.pl

**Keywords:** tensor product structures, modular observables, Fock spaces, Bell theorem, Bell inequality, quantum logic gates, classical emulation of quantum computation, Brandt-Greenberg representation

## Abstract

Any single system whose space of states is given by a separable Hilbert space is automatically equipped with infinitely many hidden tensor-like structures. This includes all quantum mechanical systems as well as classical field theories and classical signal analysis. Accordingly, systems as simple as a single one-dimensional harmonic oscillator, an infinite potential well, or a classical finite-amplitude signal of finite duration can be decomposed into an arbitrary number of subsystems. The resulting structure is rich enough to enable quantum computation, violation of Bell’s inequalities, and formulation of universal quantum gates. Less standard quantum applications involve a distinction between position and hidden position. The hidden position can be accompanied by a hidden spin, even if the particle is spinless. Hidden degrees of freedom are, in many respects, analogous to modular variables. Moreover, it is shown that these hidden structures are at the roots of some well-known theoretical constructions, such as the Brandt–Greenberg multi-boson representation of creation–annihilation operators, intensively investigated in the context of higher-order or fractional-order squeezing. In the context of classical signal analysis, the discussed structures explain why it is possible to emulate a quantum computer by classical analog circuit devices.

## 1. Introduction

Quantum computation [1,2,3,4] begins with a single quantum digit, represented by a vector |n〉, n∈{0,…,N−1}, an element of an orthonormal basis in some *N*-dimensional Hilbert space. Typical quantum digits, the qubits, correspond to N=2 and are modeled by polarization degrees of photons, spins of electrons, or states of a two-level atom. Larger values of *N* are often realized in practice by means of multi-port interferometers. In order to process more than one digit, one typically considers quantum registers consisting of multi-particle quantum systems. The mathematical structure that allows one to combine *K* quantum *N*-dimensional digits into a single register is given by a tensor product:

(1) $$\begin{array}{ccc} |l_{K-1}\ldots l_{0}\rangle & = & |l_{K-1}\rangle ⊗\cdots ⊗|l_{0}\rangle . \end{array}$$

This is how one introduces the so-called computational-basis representation of a number [5]:

(2) $$\begin{array}{c} n=N^{K-1}l_{K-1}+\cdots +N^{1}l_{1}+N^{0}l_{0}. \end{array}$$

Tensor-product bases (1) are characterized by scalar products of the form

(3) $$\begin{array}{c} \langle l_{K-1}^{\prime }\ldots l_{0}^{\prime }|l_{K-1}\ldots l_{0}\rangle =\delta_{l_{K-1}^{\prime },l_{K-1}}\ldots \delta_{l_{0}^{\prime },l_{0}}, \end{array}$$

which means that different numbers correspond to orthogonal vectors, and two numbers are different if they differ in at least one digit.

A general *K*-digit quantum state is represented by a general superposition:

(4) $$\begin{array}{ccc} |\psi \rangle & = & \sum_{l_{K-1}\ldots l_{0}=0}^{N-1}\psi_{l_{K-1}\ldots l_{0}}|l_{K-1}\ldots l_{0}\rangle . \end{array}$$

Vector |ψ〉 is said to be a product state if the amplitude $\psi_{l_{K-1}\ldots l_{0}}=f_{l_{K-1}\ldots l_{L}}g_{l_{L-1}\ldots l_{0}}$ is a product of at least two amplitudes. We then write ψ=f⊗g. Non-product |ψ〉 are called entangled. The distinction between product and entangled states occurs at the level of probability amplitudes $\psi_{l_{K-1}\ldots l_{0}}$, and not at the level of the computational-basis vectors, which are non-entangled by construction.

Products of amplitudes occur also in probability amplitudes for paths in interferometers, sometimes termed the histories, so they can be easily confused with hidden tensor products, the subject of the present paper. The distinction is most easily explained by the Kronecker product of matrices, a matrix form of a tensor product. The four-index tensor $X_{ab}Y_{cd}=Z_{abcd}$ is a matrix element of the Kronecker product X⊗Y=Z. A path occurs if b=c, so this is a three-index object, $X_{aj}Y_{jd}=Z_{ajd}$, representing a process a→j→d (or d→j→a), joining *a* with *d* and passing through *j* (hence the name path or history). If we additionally sum over the intermediate states, we obtain a transvection $\sum_{j}X_{aj}Y_{jd}=Z_{ad}$. The transvection, when interpreted in terms of paths, is equivalent to interference. Each transvection removes a pair of indices. An example of a transvection is given by a matrix product of two matrices, XY=Z. A path or history is thus an intermediate concept, halfway between tensor and matrix products.

The above brief introduction illustrates several important conceptual ingredients in the formalism of quantum computation. First of all, one thinks in terms of a hardware whose building block is a *K*-digit quantum register. Secondly, one works from the outset with finite-dimensional Hilbert spaces which, however, cannot occur in practice. And indeed, there are no true *N*-level atoms—all atoms involve infinitely many energy levels. Even objects as elementary as photons or electrons are described by infinitely dimensional Hilbert spaces. All elementary particles involve Hilbert spaces of square-integrable functions, and all such spaces are separable [6]. The latter means that one can always introduce a countable basis |n〉, indexed by integer or natural numbers. The basis is countable, even in the case where continuous degrees of freedom are present.

The goal of this paper is to show that any separable Hilbert space is naturally equipped with infinitely many hidden tensor-product structures. As opposed to the ‘bottom-up’ tensor structures (1) that demand large numbers *K* of elementary systems, we are interested in the ‘top-down’ tensor structure inherent in any single quantum system, even as simple as a one-dimensional harmonic oscillator. One should not confuse the resulting structure with the one of interfering paths, an intrinsic feature of any quantum dynamics.

At the present stage, we are more interested in the issue of fundamental principles than in their implementation in practice. However, as a by-product of our discussion, we show that hidden tensor structures are in fact sometimes literally hiding behind some well-known quantum-mechanical or quantum-like constructions. The problem of multi-photon squeezed states provides an example.

On the implementation side, our approach explains why it is possible to emulate a quantum computer by means of classical analog circuit devices [7,8,9].

## 2. Hidden Tensor Products

To begin with, let N∈N be a natural number. Any integer *n* can be uniquely written as

(5) $$\begin{array}{ccc} n & = & Nk+l,\;l=0,1,\ldots ,N-1. \end{array}$$

Here, k=⌊n/N⌋ and *l* is the remainder of the division n/N. The map

(6) $$\begin{array}{ccc} n\mapsto (k,l) & = & \left(\lfloor n/N\rfloor ,n-N\lfloor n/N\rfloor \right) \end{array}$$

(7) $$\begin{array}{ccc} & = & \left(\lfloor n/N\rfloor ,n\mathrm{mod}N\right) \end{array}$$

is one-to-one. The latter means that n=Nk+l equals $n^{\prime }=Nk^{\prime }+l^{\prime }$ if and only if $k=k^{\prime }$ and $l=l^{\prime }$. And, vice versa, $n\neq n^{\prime }$ if and only if either $k\neq k^{\prime }$ or $l\neq l^{\prime }$. What makes these trivial observations important from our point of view is the following orthonormality property of the basis vectors in our separable Hilbert space:

(8) $$\begin{array}{c} \delta_{n,n^{\prime }}=\langle n|n^{\prime }\rangle =\langle Nk+l|Nk^{\prime }+l^{\prime }\rangle =\delta_{k,k^{\prime }}\delta_{l,l^{\prime }}, \end{array}$$

a formula typical of a tensor-product structure (3). Actually, the basis |n〉, if parametrized as

(9) $$\begin{array}{c} |n\rangle =|Nk+l\rangle =|k,l\rangle , \end{array}$$

possesses the required properties of a basis in a tensor-product space.

Formula (9) is central to the paper.

The procedure can be iterated: $k=Nk_{1}+l_{1}$, etc. (which is essentially the Euclidean algorithm for the greatest common divisor), leading to

(10) $$\begin{array}{ccc} |n\rangle & = & |N^{M}k+N^{M-1}l_{M-1}+\ldots ,N^{1}l_{1}+N^{0}l_{0}\rangle \end{array}$$

(11) $$\begin{array}{ccc} & = & |k,l_{M-1},\ldots ,l_{1},l_{0}\rangle , \end{array}$$

with integer *k* and $l_{j}\in \left\{0,\ldots ,N-1\right\}$. Notice that the parametrization we use,

(12) $$\begin{array}{c} n=N^{M}k+N^{M-1}l_{M-1}+\ldots ,N^{1}l_{1}+N^{0}l_{0}, \end{array}$$

implies that the value of *M* is the same for all integers *n*. Thus, this is not exactly the usual representation of a number *n* by its digits because *k* can be arbitrarily large or negative.

For arbitrary fixed *M*, we find a tensor-product orthonormality condition:

(13) $$\begin{array}{ccc} \langle n|n^{\prime }\rangle & = & \langle k_{M},l_{M-1},\ldots ,l_{0}|k_{M}^{\prime },l_{M-1}^{\prime },\ldots ,l_{0}^{\prime }\rangle \\ & = & \delta_{k_{M},k_{M}^{\prime }}\delta_{l_{M-1},l_{M-1}^{\prime }}\ldots \delta_{l_{0},l_{0}^{\prime }}=\delta_{n,n^{\prime }}, \end{array}$$

where the *k*s are integers and $0\leq l_{j}<M$.

However, one should bear in mind that, as a consequence of (10) and (11), we can find

(14) $$\begin{array}{c} |k_{M},l_{M-1},\ldots ,l_{1},l_{0}\rangle =|k_{L},l_{L-1},\ldots ,l_{1},l_{0}\rangle , \end{array}$$

for L≠M, and, thus, (13) does not apply to

$$\begin{array}{c} \langle k_{M},l_{M-1},\ldots ,l_{0}|k_{L}^{\prime },l_{L-1}^{\prime },\ldots ,l_{0}^{\prime }\rangle \end{array}$$

if L≠M. In other words, one cannot treat the index *M* in $|k_{M},l_{M-1},\ldots ,l_{1},l_{0}\rangle$ as a tensor power in the sense we know from Fock spaces.

Rather, we should treat *k* as an external degree of freedom (such as position or momentum), and $l_{j}$ as internal degrees of freedom (such as spin). There is some similarity between *k* and $l_{j}$ and the modular variables [10,11,12,13,14,15].

However, for any *n*, there exists a maximal *M*, such that

(15) $$\begin{array}{c} \left|n\rangle =\right|l_{M-1},\ldots ,l_{1},l_{0}\rangle . \end{array}$$

The *l*s in (15) are the digits of *n*. Then, the following variant of (13) holds:

(16) $$\begin{array}{ccc} \langle n|n^{\prime }\rangle & = & \langle l_{M-1},\ldots ,l_{0}|l_{M^{\prime }-1}^{\prime },\ldots ,l_{0}^{\prime }\rangle \\ & = & \left\{\begin{array}{cc} 0 & \mathrm{if}\;M\neq M^{\prime }\\ \delta_{l_{M-1},l_{M-1}^{\prime }}\ldots \delta_{l_{0},l_{0}^{\prime }} & \mathrm{if}\;M=M^{\prime } \end{array}\right. \end{array}$$

Orthonormality (16) is typical in Fock spaces.

We conclude that hidden tensor products can be regarded as Fock-type ones if one parametrizes (non-negative) integers by their digits. If, instead, we prefer the ‘modular’ version with a fixed *M*, then the first index, $k_{M}$, is an integer. Thus, in order to distinguish between the two hidden tensor structures, we speak of modular hidden products, if we work with (10) and (11), and Fockian hidden products, if we employ (15).

Let us finally illustrate, using a concrete example, the dependence of entanglement on *N*.

An example of a product state for N=3 is, say,

(17) $$\begin{array}{ccc} |f⊗g\rangle & = & \frac{1}{\sqrt{3}}\left(|15\rangle +|16\rangle +|17\rangle \right) \end{array}$$

(18) $$\begin{array}{ccc} & = & f_{5}g_{0}|5,0\rangle +f_{5}g_{1}|5,1\rangle +f_{5}g_{2}|5,2\rangle \end{array}$$

with $f_{5}=1$, $g_{0}=g_{1}=g_{2}=1/\sqrt{3}$. Indeed, |5,0〉=|3·5+0〉, |5,1〉=|3·5+1〉, |5,2〉=|3·5+2〉. However,

(19) $$\begin{array}{ccc} |\psi \rangle & = & \frac{1}{\sqrt{2}}\left(|17\rangle +|18\rangle \right) \end{array}$$

(20) $$\begin{array}{ccc} & = & \psi_{5,2}|5,2\rangle +\psi_{6,0}|6,0\rangle \end{array}$$

is entangled (non-product), as it cannot be written as $\sum_{kl}f_{k}g_{l}|k,l\rangle$. The same state,

(21) $$\begin{array}{ccc} |\psi \rangle & = & \frac{1}{\sqrt{2}}\left(|17\rangle +|18\rangle \right) \end{array}$$

(22) $$\begin{array}{ccc} & = & \psi_{1,7}|1,7\rangle +\psi_{1,8}|1,8\rangle , \end{array}$$

is a product state if we choose N=10. Equations (20) and (22) represent the same state ψ but are decomposed into different subsystems because two different forms of a tensor product are employed. The resulting multitude of hidden tensor structures is reminiscent of the better-known bottom-up alternative tensor-product structures [16,17].

## 3. Associativity vs. Nesting

Modular hidden products are non-associative. Namely, if we write

(23) $$\begin{array}{c} |N^{2}k+Nl_{1}+l_{0}\rangle =|k,l_{1},l_{0}\rangle =|k\rangle ⊗|l_{1}\rangle ⊗|l_{0}\rangle , \end{array}$$

we obtain

(24) $$\begin{array}{c} \left(\left|k\rangle ⊗\right|l_{1}\rangle \right)⊗|l_{0}\rangle \neq |k\rangle ⊗\left(|l_{1}\rangle ⊗|l_{0}\rangle \right). \end{array}$$

Indeed,

(25) $$\begin{array}{ccc} \left(\left|k\rangle ⊗\right|l_{1}\rangle \right)⊗|l_{0}\rangle & = & |Nk+l_{1}\rangle ⊗|l_{0}\rangle \\ & = & |N\left(Nk+l_{1}\right)+l_{0}\rangle , \end{array}$$

whereas

(26) $$\begin{array}{ccc} |k\rangle ⊗\left(|l_{1}\rangle ⊗|l_{0}\rangle \right) & = & |k\rangle ⊗|Nl_{1}+l_{0}\rangle \end{array}$$

(27) $$\begin{array}{ccc} & = & |Nk+Nl_{1}+l_{0}\rangle . \end{array}$$

Actually, the very form in (26) is inconsistent because $Nl_{1}+l_{0}$ is not limited from above by N−1 and, thus, cannot be treated as an *l* variable.

However, the product possesses a nesting property which allows for an analogue of tensor multiplication, namely,

(28) $$\begin{array}{c} \left|n\rangle =\right|k_{0},l_{0}\rangle =|k_{1},l_{1},l_{0}\rangle =|k_{2},l_{2},l_{1},l_{0}\rangle =\ldots \end{array}$$

Here, the indices $l_{j}$ do not change their values as we switch between the alternative forms. In quantum mechanical notation, we could write them in a nested form:

(29) $$\begin{array}{c} |k_{0}\rangle |l_{0}\rangle =\left(|k_{1}\rangle |l_{1}\rangle \right)|l_{0}\rangle =\left(\left(|k_{2}\rangle |l_{2}\rangle \right)|l_{1}\rangle \right)|l_{0}\rangle =\ldots \end{array}$$

still maintaining the fundamental property of tensor products:

(30) $$\begin{array}{c} \left(\langle k_{1}|\langle l_{1}|\right)\langle l_{0}|\left(|k_{1}^{\prime }\rangle |l_{1}^{\prime }\rangle \right)|l_{0}^{\prime }\rangle =\delta_{k_{1},k_{1}^{\prime }}\delta_{l_{1},l_{1}^{\prime }}\delta_{l_{0},l_{0}^{\prime }}. \end{array}$$

The braces can be skipped, but we have to remember the rule in (30) that determines the order of multiplications:

(31) $$\begin{array}{c} \left(\langle k_{1}|\langle l_{1}|\langle l_{0}|\right)\left(|k_{1}^{\prime }\rangle |l_{1}^{\prime }\rangle |l_{0}^{\prime }\rangle \right)=\delta_{k_{1},k_{1}^{\prime }}\delta_{l_{1},l_{1}^{\prime }}\delta_{l_{0},l_{0}^{\prime }}. \end{array}$$

The form in (31) is indistinguishable from the standard quantum notation.

## 4. Hidden Subsystems

A subsystem is defined by specifying the form of subsystem observables: the ‘left’ subsystem observable,

(32) $$\begin{array}{ccc} Q⊗I & = & \sum_{k,k^{\prime },l}|k,l\rangle Q_{k,k^{\prime }}\langle k^{\prime },l| \end{array}$$

(33) $$\begin{array}{ccc} & = & \sum_{k,k^{\prime },l}|Nk+l\rangle Q_{k,k^{\prime }}\langle Nk^{\prime }+l|, \end{array}$$

and the ‘right’ subsystem observable,

(34) $$\begin{array}{ccc} I⊗R & = & \sum_{k,l,l^{\prime }}|k,l\rangle R_{l,l^{\prime }}\langle k,l^{\prime }| \end{array}$$

(35) $$\begin{array}{ccc} & = & \sum_{k,l,l^{\prime }}|Nk+l\rangle R_{l,l^{\prime }}\langle Nk+l^{\prime }|. \end{array}$$

The so-called reduced density matrices are a consequence of (32) and (34).

In the next section, we show that hidden tensor products indeed hide behind certain well-known theoretical constructions.

## 5. BG Representation

Hidden tensor structures imply that one can define hidden subsystems of any quantum system. In particular, the hidden tensor structure

(36) $$\begin{array}{ccc} |n\rangle & = & \frac{1}{\sqrt{n!}}a^{†n}|0\rangle =|Nk+l\rangle \end{array}$$

(37) $$\begin{array}{ccc} & = & |k,l\rangle , \end{array}$$

of a one-dimensional harmonic oscillator admits subsystem observables of the form $Q⊗I_{N}$ and $I_{\infty }⊗R$.

In order to make this statement more formal, let us consider two vectors,

(38) $$\begin{array}{ccc} |f\rangle & = & \sum_{k=0}^{\infty }f_{k}|k\rangle \in H_{\infty }, \end{array}$$

(39) $$\begin{array}{ccc} |g\rangle & = & \sum_{l=0}^{N-1}g_{l}|l\rangle \in H_{N}, \end{array}$$

in some Hilbert spaces of appropriate dimensions. The hidden tensor product is defined by

(40) $$\begin{array}{ccc} |f⊗g\rangle & = & \sum_{k=0}^{\infty }\sum_{l=0}^{N-1}f_{k}g_{l}|k,l\rangle \end{array}$$

(41) $$\begin{array}{ccc} & = & \sum_{k=0}^{\infty }\sum_{l=0}^{N-1}f_{k}g_{l}|Nk+l\rangle \end{array}$$

(42) $$\begin{array}{ccc} & = & \sum_{n=0}^{\infty }f_{\left\lfloor n/N\right\rfloor }g_{n-N\lfloor n/N\rfloor }|n\rangle \end{array}$$

In this way, the Hilbert space H of a one-dimensional harmonic oscillator becomes a tensor-product Hilbert space $H_{\infty }⊗H_{N}$ with the basis |k,l〉. Let us stress that we have not introduced a new Hilbert space because $H=H_{\infty }⊗H_{N}$. This is still the same textbook Hilbert space of the one-dimensional harmonic oscillator, which should be clear from (40)–(42).

The creation operator in $H_{\infty }$ can be defined in the usual way:

(43) $$\begin{array}{ccc} b^{†} & = & \sum_{k=0}^{\infty }\sqrt{k+1}\left|k+1\rangle \langle k\right| \end{array}$$

(we distinguish between $b^{†}$, which acts in $H_{\infty }$, and the ordinary textbook $a^{†}$ that acts in $H=H_{\infty }⊗H_{N}$). This is a typical subsystem operator, related to subsystem canonical position $X_{\infty }$ and momentum $P_{\infty }$ by $X_{\infty }∼b+b^{†}$, $P_{\infty }∼i\left(b^{†}-b\right)$.

Now, let us consider its hidden tensor product with the identity operator $I_{N}$ in $H_{N}$:

(44) $$\begin{array}{ccc} A_{N}^{†} & = & b^{†}⊗I_{N}, \end{array}$$

(45) $$\begin{array}{ccc} A_{N} & = & b⊗I_{N}, \end{array}$$

(46) $$\begin{array}{ccc} A_{N}^{†}|f⊗g\rangle & = & |b^{†}f⊗g\rangle \end{array}$$

Obviously, $A_{N}$ and $A_{N}^{†}$ operate in H, and

(47) $$\begin{array}{c} \left[A_{N},A_{N}^{†}\right]=I_{\infty }⊗I_{N}=I=\left[a,a^{†}\right]. \end{array}$$

Contrary to appearances, one should not automatically identify $A_{N}$ with *a*. Let us, for the moment, forget about the subtleties with associativity and nesting, whose analysis we have shifted to Appendix A, and perform the following calculation:

(48) $$\begin{array}{c} A_{NN^{\prime }}=b⊗I_{NN^{\prime }}=b⊗I_{N}⊗I_{N^{\prime }}=A_{N}⊗I_{N^{\prime }}. \end{array}$$

Its result is a formula typical of Brandt–Greenberg (BG) *N*-boson creation–annihilation operators [18]. However, as known from the literature, the relationship between the BG operators and the ordinary *a* and $a^{†}$ is much more complicated:

(49) $$\begin{array}{ccc} A_{N} & = & a^{N}\sqrt{\left\lfloor a^{†}a/N\right\rfloor \left(a^{†}a-N\right)!/\left(a^{†}a\right)!} \end{array}$$

(50) $$\begin{array}{ccc} & = & F_{N}\left(a,a^{†}\right), \end{array}$$

and does not resemble (45) in the least. On the other hand, one indeed finds [19]

(51) $$\begin{array}{c} F_{NN^{\prime }}\left(a,a^{†}\right)=F_{N^{\prime }}\left(A_{N},A_{N}^{†}\right), \end{array}$$

but its proof based on (49) is far less evident than the trivial calculation from (48).

Furthermore, note that the forms in (44) and (45) are trivially normally ordered, whereas the simplest normally ordered form of the BG operator $A_{N}$ one finds in the literature is

(52) $$\begin{array}{ccc} A_{N} & = & \sum_{j=0}^{\infty }\alpha_{j}^{\left(N\right)}a^{†j}a^{j+N}, \end{array}$$

(53) $$\begin{array}{ccc} \alpha_{j}^{\left(N\right)} & = & \sum_{l=0}^{j}\frac{{\left(-1\right)}^{j-l}}{(j-l)!}\sqrt{\frac{1+\lfloor l/N\rfloor }{l!(l+N)!}} \end{array}$$

Expressions (45) and (49) are so different that one may really have doubts if we have not abused notation by denoting both operators by the same symbol.

Yet, we will now directly show that we have not abused notation—both forms of $A_{N}$ represent the same operator. $A_{N}$ looks so simple if one realizes that this is a tensor-product operator, but with respect to the hidden tensor structure, implicit in the separable Hilbert space of a one-dimensional harmonic oscillator.

By definition of ⊗ and |k,l〉, formula (44) implies that

(54) $$\begin{array}{ccc} A_{N}^{†} & = & \sum_{k=0}^{\infty }\sum_{l=0}^{N-1}\sqrt{k+1}\left|k+1,l\rangle \langle k,l\right| \end{array}$$

(55) $$\begin{array}{ccc} & = & \sum_{k=0}^{\infty }\sum_{l=0}^{N-1}\sqrt{k+1}|N\left(k+1\right)+l\rangle \langle Nk+l|. \end{array}$$

It is clear from (55) that the only non-zero matrix elements of $A_{N}^{†}$ are $\langle n+N|A_{N}^{†}|n\rangle$ for some *n*. Because of the one-to-one property of the map n↦(k,l), we can replace the double sum in (55) by a single sum over *n*. It is simplest to directly evaluate the matrix element

(56) $$\begin{array}{cc} \langle n & +\;N|A_{N}^{†}|n\rangle \\ & =\sum_{k=0}^{\infty }\sum_{l=0}^{N-1}\sqrt{k+1}\langle n+N|Nk+l+N\rangle \langle Nk+l|n\rangle . \end{array}$$

The only non-vanishing term in the double sum corresponds to n=Nk+l. The inverse map implies k=⌊n/N⌋, l=n−N⌊n/N⌋, so that

(57) $$\begin{array}{ccc} \langle n+N|A_{N}^{†}|n\rangle & = & \sqrt{\lfloor n/N\rfloor +1}, \end{array}$$

and

(58) $$\begin{array}{ccc} A_{N}^{†} & = & \sum_{n=0}^{\infty }\sqrt{\left(\lfloor n/N\rfloor +1\right)}\left|n+N\rangle \langle n\right| \end{array}$$

(59) $$\begin{array}{ccc} & = & \sqrt{\left\lfloor \hat{n}/N\right\rfloor \left(\hat{n}-N\right)!/\hat{n}!}{\left(a^{†}\right)}^{N}, \end{array}$$

which is indeed the BG operator. One concludes that the BG creation operator is the usual creation operator $b^{†}$, only operating in the ‘left’ subsystem defined by |k,l〉=|Nk+l〉. Alternatively, one can directly write

(60) $$\begin{array}{ccc} |k,l\rangle & = & \frac{1}{\sqrt{(Nk+l)!}}a^{†Nk+l}|0\rangle , \end{array}$$

(61) $$\begin{array}{ccc} |k,0\rangle & = & \frac{1}{\sqrt{(Nk)!}}a^{†Nk}|0,0\rangle \end{array}$$

(62) $$\begin{array}{ccc} & = & \frac{1}{\sqrt{k!}}b^{†k}⊗I_{N}|0,0\rangle \end{array}$$

(63) =1k!AN†|k0〉.

From a purely conceptual point of view, it is perhaps even more important that it be allowed to contradict the whole tradition of research on BG operators and treat $A_{N}$ just as a form of *a*, that is, begin with the hidden tensor structure defined by $a=b⊗I_{N}$ and |n〉=|Nk+l〉=|k〉⊗|l〉.

BG coherent states were intensively investigated in the 1980s as a possible alternative to *N*-boson squeezed-states $e^{za^{†N}-\bar{z}a^{N}}|0\rangle$. The latter are mathematically problematic [20,21], whereas $e^{zA_{N}^{†}-\bar{z}A_{N}}|0\rangle$ are as well-behaved as the ordinary coherent states in H. The N=2 case of $e^{zA_{N}^{†}-\bar{z}A_{N}}|0\rangle$ indeed leads to squeezing of variances of position $X∼a+a^{†}$ and momentum $P∼i(a^{†}-a)$, although qualitatively different from the squeezing implied by $e^{za^{†2}-\bar{z}a^{2}}|0\rangle$ [22].

A general state representing the hidden subsystem associated with $H_{\infty }$ can be described by means of its reduced density matrix $\rho_{\infty }$. To this end, consider a general state $|\psi \rangle \in H=H_{\infty }⊗H_{N}$,

(64) $$\begin{array}{c} |\psi \rangle =\sum_{n}\psi_{n}|n\rangle =\sum_{k,l}\psi_{Nk+l}|Nk+l\rangle =\sum_{k,l}\psi_{k,l}|k,l\rangle . \end{array}$$

By definition,

(65) $$\begin{array}{ccc} {\left(\rho_{\infty }\right)}_{k,k^{\prime }} & = & \sum_{l=0}^{N-1}\psi_{k,l}\bar{\psi_{k^{\prime },l}}=\sum_{l=0}^{N-1}\psi_{Nk+l}\bar{\psi_{Nk^{\prime }+l}} \end{array}$$

(66) $$\begin{array}{ccc} & = & \sum_{l=0}^{N-1}\psi_{N\lfloor n/N\rfloor +l}\bar{\psi_{N\lfloor n^{\prime }/N\rfloor +l}}. \end{array}$$

Reduced density matrix $\rho_{N}$ is defined analogously,

(67) $$\begin{array}{ccc} {\left(\rho_{N}\right)}_{l,l^{\prime }} & = & \sum_{k=0}^{\infty }\psi_{k,l}\bar{\psi_{k,l^{\prime }}}=\sum_{k=0}^{\infty }\psi_{Nk+l}\bar{\psi_{Nk+l^{\prime }}}. \end{array}$$

## 6. Hidden Statistics of a Coherent State

### 6.1. Two Hidden Subsystems

Consider the coherent state satisfying a|z〉=z|z〉:

(68) $$\begin{array}{ccc} |z\rangle & = & e^{-{\left|z\right|}^{2}/2}\sum_{n=0}^{\infty }\frac{z^{n}}{\sqrt{n!}}|n\rangle =\sum_{k,l}\psi_{Nk+l}|Nk+l\rangle \end{array}$$

Its hidden statistics of excitations are determined by the diagonal matrix element ${\left(\rho_{\infty }\right)}_{k,k}$. For N=1, ${\left(\rho_{\infty }\right)}_{k,k}$ is just the coherent-state Poisson distribution. For N=2,

(69) $$\begin{array}{c} {\left(\rho_{\infty }\right)}_{k,k}=e^{-{\left|z\right|}^{2}}\left(\frac{{\left(|z\right|}^{2}{)}^{2k}}{(2k)!}+\frac{{\left(|z\right|}^{2}{)}^{2k+1}}{(2k+1)!}\right) \end{array}$$

describes the probability of finding excitations within the the interval [2k,2k+1]. For N=3,

(70) $$\begin{array}{c} {\left(\rho_{\infty }\right)}_{k,k}=e^{-{\left|z\right|}^{2}}\left(\frac{{\left(|z\right|}^{2}{)}^{3k}}{(3k)!}+\frac{{\left(|z\right|}^{2}{)}^{3k+1}}{(3k+1)!}+\frac{{\left(|z\right|}^{2}{)}^{3k+2}}{(3k+2)!}\right), \end{array}$$

is an analogous probability for [3k,3k+2]. A generalization to arbitrary *N* is now obvious.

Statistics for the ‘right’, *N*-dimensional subsystem appear as follows:

(71) $$\begin{array}{c} {\left(\rho_{N}\right)}_{l,l}=e^{-{\left|z\right|}^{2}}\sum_{k=0}^{\infty }\frac{{\left(|z\right|}^{2}{)}^{Nk+l}}{(Nk+l)!}, \end{array}$$

so this is the probability of finding an *n*th excitation with n≡lmodN. Formulas (69)–(71) provide an operational definition of the hidden subsystems.

### 6.2. Three Hidden Subsystems

The three-subsystem decomposition of the harmonic-oscillator Hilbert space is obtained by means of

(72) $$\begin{array}{c} n=Nk_{0}+l_{0}=N\left(Nk_{1}+l_{1}\right)+l_{0} \end{array}$$

and

(73) $$\begin{array}{c} \left|n\rangle =\right|k_{1},l_{1},l_{0}\rangle ,\;k_{1}\in Z_{+},\;l_{1},l_{0}\in \left\{0,\ldots ,N-1\right\}. \end{array}$$

The map $n\mapsto (k_{1},l_{1},l_{0})$ is one-to-one as a composition of two one-to-one maps: $n\mapsto (k_{0},l_{0})$ and $k_{0}\mapsto \left(k_{1},l_{1}\right)$. The three reduced density matrices read

(74) $$\begin{array}{ccc} {\left(\rho_{\infty }\right)}_{k_{1},k_{1}^{\prime }} & = & \sum_{l_{1},l_{0}}\psi_{N^{2}k_{1}+Nl_{1}+l_{0}}\bar{\psi_{N^{2}k_{1}^{\prime }+Nl_{1}+l_{0}}}, \end{array}$$

(75) $$\begin{array}{ccc} {\left(\rho_{N_{1}}\right)}_{l_{1},l_{1}^{\prime }} & = & \sum_{k_{1},l_{0}}\psi_{N^{2}k_{1}+Nl_{1}+l_{0}}\bar{\psi_{N^{2}k_{1}+Nl_{1}^{\prime }+l_{0}}}, \end{array}$$

(76) $$\begin{array}{ccc} {\left(\rho_{N_{0}}\right)}_{l_{0},l_{0}^{\prime }} & = & \sum_{k_{1},l_{1}}\psi_{N^{2}k_{1}+Nl_{1}+l_{0}}\bar{\psi_{N^{2}k_{1}+Nl_{1}+l_{0}^{\prime }}}. \end{array}$$

For (68) and N=2, we find

(77) $$\begin{array}{ccc} {\left(\rho_{\infty }\right)}_{k,k} & = & e^{-{\left|z\right|}^{2}}\sum_{l_{1},l_{0}=0}^{1}\frac{{\left(|z\right|}^{2}{)}^{2^{2}k+2l_{1}+l_{0}}}{(2^{2}k+2l_{1}+l_{0})!} \end{array}$$

(78) $$\begin{array}{ccc} & = & e^{-{\left|z\right|}^{2}}\sum_{l=0}^{3}\frac{{\left(|z\right|}^{2}{)}^{4k+l}}{(4k+l)!}. \end{array}$$

Comparison with (69) and (70) shows that probabilities (78) could have also been obtained directly from the two-subsystem case N=4, but the remaining two hidden-subsystem single-qubit reduced-density matrices would have no counterpart for N=4. Here, for N=2 and the three-subsystem decomposition, we obtain

(79) $$\begin{array}{ccc} \rho_{N_{1}} & = & e^{-{\left|z\right|}^{2}}\sum_{k,l}\frac{{\left|z\right|}^{2(4k+l)}}{(4k+l)!}\\ & \times & \left(\begin{array}{cc} 1 & \frac{{\bar{z}}^{2}}{\sqrt{\left(4k+l+1)(4k+l+2\right)}}\\ \frac{z^{2}}{\sqrt{\left(4k+l+1)(4k+l+2\right)}} & \frac{{\left|z\right|}^{4}}{\left(4k+l+1)(4k+l+2\right)} \end{array}\right), \end{array}$$

(80) $$\begin{array}{ccc} \rho_{N_{0}} & = & e^{-{\left|z\right|}^{2}}\sum_{k,l}\frac{{\left|z\right|}^{2(4k+2l)}}{(4k+2l)!}\left(\begin{array}{cc} 1 & \frac{\bar{z}}{\sqrt{4k+2l+1}}\\ \frac{z}{\sqrt{4k+2l+1}} & \frac{{\left|z\right|}^{2}}{4k+2l+1} \end{array}\right). \end{array}$$

The above two reduced 2×2 single-qubit density matrices represent states of two single-qubit hidden subsystems of a one-dimensional harmonic oscillator in a coherent state |z〉. Such a formal structure has no counterpart in the usual textbook presentation of a quantum harmonic oscillator.

## 7. Position vs. Hidden Position

For N=2, we naturally obtain an analogue of a spinor structure, even if the particle in question is spinless and one-dimensional:

(81) $$\begin{array}{ccc} |\psi \rangle & = & \sum_{n}\psi_{n}|n\rangle =\sum_{k}\sum_{l=0}^{1}\psi_{2k+l}|2k+l\rangle \end{array}$$

(82) $$=\underset{|\psi_{0}\rangle }{\underset{︸}{\sum_{k}\psi_{k,0}|k,0\rangle }}+\underset{|\psi_{1}\rangle }{\underset{︸}{\sum_{k}\psi_{k,1}|k,1\rangle }}.$$

Since

(83) $$\begin{array}{c} \langle k,0|k^{\prime },1\rangle =\langle 2k|2k^{\prime }+1\rangle =0, \end{array}$$

we find $\langle \psi_{0}|\psi_{1}\rangle =0$ and

(84) $$\begin{array}{c} 1=\langle \psi |\psi \rangle =\langle \psi_{0}|\psi_{0}\rangle +\langle \psi_{1}|\psi_{1}\rangle . \end{array}$$

In position representation,

(85) $$\begin{array}{c} \langle \psi_{l}|\psi_{l}\rangle =\int dx\;{\left|\psi_{l}\left(x\right)\right|}^{2}, \end{array}$$

a formula that allows us to discuss probability of hidden spin and probability density of hidden position, namely,

(86) $$\begin{array}{ccc} p_{l} & = & \int dx\;|\psi_{l}{\left(x\right)|}^{2}, \end{array}$$

(87) $$\begin{array}{ccc} \rho_{\mathrm{hid}}\left(x\right) & = & |\psi_{0}{\left(x\right)|}^{2}+{\left|\psi_{1}\left(x\right)\right|}^{2}, \end{array}$$

where

(88) $$\begin{array}{ccc} \psi_{l}\left(x\right) & = & \langle x|\psi_{l}\rangle =\sum_{k}\psi_{k,l}\langle x|k,l\rangle \end{array}$$

(89) $$\begin{array}{ccc} & = & \sum_{k}\psi_{2k+l}\langle x|2k+l\rangle \end{array}$$

(90) $$\begin{array}{ccc} & = & \sum_{k}\langle x|2k+l\rangle \langle 2k+l|\psi \rangle . \end{array}$$

Resolution of unity implies

(91) $$\begin{array}{ccc} \psi_{0}\left(x\right)+\psi_{1}\left(x\right) & = & \sum_{k,l}\langle x|2k+l\rangle \langle 2k+l|\psi \rangle \end{array}$$

(92) $$\begin{array}{ccc} & = & \sum_{n}\langle x|n\rangle \langle n|\psi \rangle =\langle x|\psi \rangle =\psi \left(x\right). \end{array}$$

We can finally pinpoint the difference between position and hidden position. Probability density of position is given by

(93) $$\begin{array}{ccc} \rho (x) & = & {\left|\psi \left(x\right)\right|}^{2}={\left|\psi_{0}\left(x\right)+\psi_{1}\left(x\right)\right|}^{2} \end{array}$$

(94) $$\begin{array}{ccc} & = & \rho_{\mathrm{hid}}\left(x\right)+2ℜ\left(\bar{\psi_{0}\left(x\right)}\psi_{1}\left(x\right)\right). \end{array}$$

The normalization is preserved:

(95) $$\begin{array}{ccc} \int dx\;\rho (x) & = & \int dx\;\rho_{\mathrm{hid}}\left(x\right)+2ℜ\underset{0}{\underset{︸}{\langle \psi_{0}|\psi_{1}\rangle }}. \end{array}$$

## 8. Parity as a Hidden Spin

In order to explicitly construct U(2) spinor transformations of the hidden spin, we define

(96) $$\begin{array}{c} \left(I⊗U\right)\psi_{k,l}=\sum_{l^{\prime }=0}^{1}U_{ll^{\prime }}\psi_{k,l^{\prime }}, \end{array}$$

where $U_{ll^{\prime }}$ is a unitary 2×2 matrix. In terms of $\psi_{n}$ the transformation reads

(97) $$\begin{array}{c} \left(I⊗U\right)\psi_{2k+l}=\sum_{l^{\prime }=0}^{1}U_{ll^{\prime }}\psi_{2k+l^{\prime }}, \end{array}$$

and

(98) $$\begin{array}{ccc} |(I⊗U)\psi \rangle & = & \sum_{k}\sum_{l=0}^{1}\left(I⊗U\right)\psi_{2k+l}|2k+l\rangle \end{array}$$

(99) $$\begin{array}{ccc} & = & \sum_{k}\sum_{l^{\prime }=0}^{1}\psi_{2k+l^{\prime }}\sum_{l=0}^{1}U_{ll^{\prime }}|2k+l\rangle \end{array}$$

(100) $$\begin{array}{ccc} & = & \sum_{k}\sum_{l=0}^{1}\psi_{2k+l}\left(I⊗U\right)|2k+l\rangle , \end{array}$$

where

(101) $$\begin{array}{ccc} (I⊗U)|2k+l\rangle & = & \sum_{l^{\prime }=0}^{1}U_{l^{\prime }l}|2k+l^{\prime }\rangle . \end{array}$$

Transformation I⊗U is indeed unitary:

$$\begin{array}{ccc} & & \langle 2k_{1}+l_{1}|{\left(I⊗U\right)}^{†}\left(I⊗U\right)|2k_{2}+l_{2}\rangle \end{array}$$

(102) $$=\sum_{l_{1}^{\prime }=0}^{1}\sum_{l_{2}^{\prime }=0}^{1}\bar{U_{l_{1}^{\prime }l_{1}}}U_{l_{2}^{\prime }l_{2}}\langle 2k_{1}+l_{1}^{\prime }|2k_{2}+l_{2}^{\prime }\rangle$$

(103) =∑l1′=01∑l2′=01U−1Ul2′l2l1l1′δk1,k2δl1′,l2′

(104) $$=\delta_{k_{1},k_{2}}\delta_{l_{1},l_{2}}=\langle 2k_{1}+l_{1}|2k_{2}+l_{2}\rangle ,$$

and acts only on the binary (spinor) indices.

The Bloch vector is defined in the usual way, as

(105) $$\begin{array}{ccc} s & = & \langle \psi |I_{\infty }⊗\sigma |\psi \rangle \end{array}$$

(106) $$\begin{array}{ccc} & = & \sum_{k}\sum_{l,l^{\prime }}\bar{\psi_{k,l}}\sigma_{ll^{\prime }}\psi_{k,l^{\prime }} \end{array}$$

(107) $$\begin{array}{ccc} & = & \sum_{k}\sum_{l,l^{\prime }}\bar{\psi_{2k+l}}\sigma_{ll^{\prime }}\psi_{2k+l^{\prime }}. \end{array}$$

As an example, consider a free one-dimensional scalar particle on the interval [−L/2,L/2], with the standard scalar product

(108) $$\begin{array}{c} \langle f|g\rangle =\int_{-L/2}^{L/2}dx\;\bar{f(x)}g\left(x\right). \end{array}$$

Let us take the basis of real standing waves:

(109) $$\begin{array}{ccc} \langle x|2j\rangle & = & \sqrt{\frac{2}{L}}\cos\frac{(2j+1)\pi x}{L}, \end{array}$$

(110) $$\begin{array}{ccc} \langle x|2j+1\rangle & = & \sqrt{\frac{2}{L}}\sin\frac{(2j+2)\pi x}{L}. \end{array}$$

For antisymmetric ψ(x), we find $\psi_{2j}=\psi_{j,0}=0$; for symmetric ψ(x), we find $\psi_{2j+1}=\psi_{j,1}=0$, so that

(111) $$\begin{array}{c} s=\left(s_{1},s_{2},s_{3}\right)=\left(0,0,\pm 1\right), \end{array}$$

where $\psi \left(-x\right)=s_{3}\psi \left(x\right)$. In this concrete basis, hidden spin one-half can be identified with parity.

Symmetric or antisymmetric ψ(x) satisfy

(112) $$\begin{array}{c} \rho_{\mathrm{hid}}\left(x\right)=\rho \left(x\right). \end{array}$$

A change of basis—for example, by selecting different boundary conditions—will rotate s on the Bloch sphere and will influence the relation between symmetry of ψ(x) and the form of $\rho_{\mathrm{hid}}\left(x\right)$.

## 9. Hidden Quantum Computation

The very idea that a subspace ($N^{K}$-dimensional, say) of a Hilbert space of a single quantum system can be used to define a *K*-digit computational basis is not in itself new [23]. However, our parametrization

(113) $$\begin{array}{c} \left|n\rangle =\right|k_{0},l_{0}\rangle =|k_{M},l_{M-1},\ldots ,l_{0}\rangle ,\;k_{M}\in Z, \end{array}$$

is not just a vector representation of *n* in terms of digits. This is especially visible in the formula

(114) $$\begin{array}{c} |n\rangle =|\lfloor n/N\rfloor \rangle ⊗|n\mathrm{mod}N\rangle , \end{array}$$

linking hidden tensor product with modular arithmetic of integers. Representation in terms of hidden tensor products links number-theoretic structures with internal symmetries in Hilbert spaces. For example, the map

(115) $$\begin{array}{c} |k,l\rangle \mapsto |k,al\mathrm{mod}N\rangle \end{array}$$

is unitary if *a* and *N* are coprime. Analogously, many theorems of classical number theory can be automatically translated into theorems about unitary transformations on tensor-product Hilbert spaces—and, perhaps, the other way around.

On the other hand, thinking in terms of tensor products is typical of many quantum algorithms, so, once we identify a hidden tensor structure, we can follow certain well-established strategies of quantum information processing.

In such a new paradigm, one begins with as many subsystem decompositions as one needs for a given task, in exact analogy to the two-subsystem and three-subsystem decompositions we have just discussed on the coherent-state example. Since the subsystems can be uniquely identified, it remains to define appropriate universal quantum gates, formulated according to the standard procedures discussed in the literature [23,24].

As an illustration of a universal quantum gate that acts on a hidden coherent-state subsystem of a one-dimensional oscillator, consider the Hadamard gates $H_{0}$ and $H_{1}$, defined by

(116) $$\begin{array}{ccc} H_{0}|l_{K-1}\ldots l_{1},0_{0}\rangle & = & \frac{1}{\sqrt{2}}|l_{K-1}\ldots l_{1},0_{0}\rangle \\ & & +\frac{1}{\sqrt{2}}|l_{K-1}\ldots l_{1},1_{0}\rangle , \end{array}$$

(117) $$\begin{array}{ccc} H_{0}|l_{K-1}\ldots l_{1},1_{0}\rangle & = & \frac{1}{\sqrt{2}}|l_{K-1}\ldots l_{1},0_{0}\rangle \\ & & -\frac{1}{\sqrt{2}}|l_{K-1}\ldots l_{1},1_{0}\rangle , \end{array}$$

and

(118) $$\begin{array}{ccc} H_{1}|l_{K-1}\ldots l_{2},0_{1},l_{0}\rangle & = & \frac{1}{\sqrt{2}}|l_{K-1}\ldots l_{2},0_{1},l_{0}\rangle \\ & & \frac{1}{\sqrt{2}}|l_{K-1}\ldots l_{2},1_{1},l_{0}\rangle \end{array}$$

(119) $$\begin{array}{ccc} H_{1}|l_{K-1}\ldots l_{2},1_{1},l_{0}\rangle & = & \frac{1}{\sqrt{2}}|l_{K-1}\ldots l_{2},0_{1},l_{0}\rangle \\ & & \frac{1}{\sqrt{2}}|l_{K-1}\ldots l_{2},1_{1},l_{0}\rangle \end{array}$$

At the level of eigenvectors of $a^{†}a$, the operators read

(120) $$\begin{array}{ccc} H_{0}|2k\rangle & = & \frac{1}{\sqrt{2}}\left(|2k\rangle +|2k+1\rangle \right), \end{array}$$

(121) $$\begin{array}{ccc} H_{0}|2k+1\rangle & = & \frac{1}{\sqrt{2}}\left(|2k\rangle -|2k+1\rangle \right), \end{array}$$

and

(122) $$\begin{array}{ccc} H_{1}|2^{2}k_{1}+l_{0}\rangle & = & \frac{1}{\sqrt{2}}\left(|2^{2}k_{1}+l_{0}\rangle +|2^{2}k_{1}+2+l_{0}\rangle \right), \end{array}$$

(123) $$\begin{array}{ccc} H_{1}|2^{2}k_{1}+2+l_{0}\rangle & = & \frac{1}{\sqrt{2}}\left(|2^{2}k_{1}+l_{0}\rangle -|2^{2}k_{1}+2+l_{0}\rangle \right). \end{array}$$

Of particular interest is the action of these two universal gates on the coherent state, a|z〉=z|z〉. One finds

(124) $$\begin{array}{c} \langle n|H_{0}|z\rangle =\frac{e^{-{\left|z\right|}^{2}/2}}{\sqrt{2}}\left\{\begin{array}{cc} \frac{z^{n}}{\sqrt{n!}}+\frac{z^{n+1}}{\sqrt{(n+1)!}} & \mathrm{for}\;n=2k,\\ \frac{z^{n-1}}{\sqrt{(n-1)!}}-\frac{z^{n}}{\sqrt{n!}} & \mathrm{for}\;n=2k+1, \end{array}\right. \end{array}$$

and

(125) $$\begin{array}{c} \langle n|H_{1}|z\rangle =\frac{e^{-{\left|z\right|}^{2}/2}}{\sqrt{2}}\left\{\begin{array}{cc} \frac{z^{n}}{\sqrt{n!}}+\frac{z^{n+2}}{\sqrt{(n+2)!}} & \mathrm{for}\;n=4k+l_{0},\\ \frac{z^{n-2}}{\sqrt{(n-2)!}}-\frac{z^{n}}{\sqrt{n!}} & \mathrm{for}\;n=4k+2+l_{0}. \end{array}\right. \end{array}$$

## 10. Hidden Entanglement

The hidden spinor structure discussed in Section 7 was constructed by means of the zeroth bit $l_{0}$. One can analogously define spins higher than one-half, or construct systems analogous to multi-particle entangled states. As our final example, let us analyze the problem of the Bell inequality [25] and its violation by hidden-spin singlet-state correlations.

Consider N=2 and the three-subsystem decomposition of the harmonic-oscillator basis:

(126) $$\begin{array}{ccc} |n\rangle & = & |2^{2}k_{1}+2l_{1}+l_{0}\rangle =|k_{1},l_{1},l_{0}\rangle . \end{array}$$

Let

(127) $$\begin{array}{ccc} |\psi \rangle & = & \frac{1}{\sqrt{2}}\sum_{k=0}^{\infty }\psi_{k}\left(|k,0_{1},1_{0}\rangle -|k,1_{1},0_{0}\rangle \right) \end{array}$$

(128) $$\begin{array}{ccc} & = & \frac{1}{\sqrt{2}}\sum_{k=0}^{\infty }\psi_{k}\left(|4k+1\rangle -|4k+2\rangle \right), \end{array}$$

for some $\psi_{k}$. The two sets of Pauli matrices, representing bits in position 1 or 0, are defined by means of the tensor-product structure in the usual way:

(129) $$I_{\infty }⊗\sigma ⊗I_{0}=\sum_{k=0}^{\infty }\sum_{l_{1},l_{1}^{\prime },l_{0}=0}^{1}|k,l_{1},l_{0}\rangle \sigma_{l_{1},l_{1}^{\prime }}\langle k,l_{1}^{\prime },l_{0}|,$$

(130) $$=\sum_{k,l_{1},l_{1}^{\prime },l_{0}}|4k+2l_{1}+l_{0}\rangle \sigma_{l_{1},l_{1}^{\prime }}\langle 4k+2l_{1}^{\prime }+l_{0}|,$$

and

(131) $$I_{\infty }⊗I_{1}⊗\sigma =\sum_{k=0}^{\infty }\sum_{l_{1},l_{0},l_{0}^{\prime }=0}^{1}|k,l_{1},l_{0}\rangle \sigma_{l_{0},l_{0}^{\prime }}\langle k,l_{1},l_{0}^{\prime }|$$

(132) $$=\sum_{k,l_{1},l_{0},l_{0}^{\prime }}|4k+2l_{1}+l_{0}\rangle \sigma_{l_{0},l_{0}^{\prime }}\langle 4k+2l_{1}+l_{0}^{\prime }|.$$

The standard calculation leads to

(133) $$\begin{array}{c} \langle \psi |I_{\infty }⊗a\cdot \sigma ⊗b\cdot \sigma |\psi \rangle =-a\cdot b. \end{array}$$

Equations (128), (130), and (132) only refer to the excited states $|n\rangle =|4k_{1}+2l_{1}+l_{0}\rangle$ of a single one-dimensional harmonic oscillator, with no ‘bottom-up’ multi-particle tensor products (1). Obviously, average (133) violates Bell-type inequalities [25], a fact once again proving that all the necessary entanglement properties are encoded in states of a single one-dimensional harmonic oscillator.

Mutatis mutandis, they are present in any quantum system described by a separable Hilbert space.

## 11. Links with Classical Emulation of Quantum Computation

The discussed structures are based on a single assumption, namely, that the space of states is given by a separable Hilbert space. This is one of the von Neumann axioms in quantum mechanics. The canonical example of a separable Hilbert space is the space of square-integrable functions, a mathematical structure shared by quantum mechanics with classical electrodynamics, acoustics, and, last but not least, classical signal analysis. Hence, this leads one to question if one can somehow implement a quantum computer by means of a completely classical signal analysis. The answer is, in principle, yes.

Actually, in a series of recent papers, La Cour and his collaborators have demonstrated that an emulation of a quantum computation by means of analog circuits is practically feasible [7,8,9]. The key element in their approach is the *octave spacing scheme*, discussed in detail in [7], which is a form of binary coding of a Fockian type, as we have discussed above. Each qubit is here represented by its carrier frequency $\omega_{c}$. If $\omega_{b}$ is a baseband offset frequency, then any $\omega_{c}=\omega_{b}+2^{j}\Delta \omega$, for some *j* and Δω>0. And this is enough, from the point of view of our analysis.

Any function of several variables, say, ψ, is formally equivalent to a tensor-product state vector |ψ〉. The link between the *state* and its *wave-function* (i.e., its amplitude) $\psi (\omega_{1},\ldots ,\omega_{n})$ is given by the Dirac recipe:

(134) $$\begin{array}{ccc} \psi (\omega_{1},\ldots ,\omega_{n}) & = & \langle \omega_{1},\ldots ,\omega_{n}|\psi \rangle . \end{array}$$

Product states correspond to functions with separable coordinates, such as

(135) $$\begin{array}{ccc} \psi (\omega_{1},\omega_{2}) & = & f\left(\omega_{1}\right)g\left(\omega_{2}\right)=\langle \omega_{1},\omega_{2}|f⊗g\rangle . \end{array}$$

Sometimes a confusion arises (cf. [7,8]) if the commutativity of function values,

(136) $$\begin{array}{c} f\left(\omega_{1}\right)g\left(\omega_{2}\right)=g\left(\omega_{2}\right)f\left(\omega_{1}\right), \end{array}$$

should be identified with commutativity of such a tensor structure, namely, if this is equivalent to |f⊗g〉=|g⊗f〉, but the answer is, of course, no. Non-commuativity of the tensor product follows here from the unique identification of $\omega_{1}$ with *f* and $\omega_{2}$ with *g*. A very similar identification of a ‘position’ in a tensor product with appropriate index is employed by Penrose in his abstract-index formalism [26,27]. As correctly noticed in [7,8], what is essential for the unique coding of a basis vector is the octave spacing that allows one to identify an *n*-tuple of frequencies with a unique *n*-bit number.

## 12. Final Remarks

According to Feynman’s famous statement, quantum mechanics is ‘a theory that no one understands’. There are two levels to this lack of understanding. One is purely physical: we just do not have everyday experiences with the microworld. The second is related to the mathematical structure of the theory—there is not even a general agreement on what should be meant by quantization, or why there is first quantization and second quantization.

Reformulation of separable Hilbert spaces in terms of hidden tensor products allows us to rephrase various standard structures in quantum mechanics. We have given several examples of such a rephrasing: parity as a form of hidden spin, hidden spin degrees of freedom of a spinless particle, GB multi-boson operators as simple hidden tensor products, quantum gates operating on hidden subsystems, or internal entanglement between hidden subsystems. There are intriguing and unexplored connections between hidden tensor structures and methods of classical number theory. Is this already a third quantization?

Furthermore, we have purposefully left open the issue of uniqueness of ⊗. Depending on *N*, the same state can be simultaneously entangled and non-entangled. This suggests nontriviality of the problem. The most important subtlety can be illustrated as follows. Assume |cyan〉, |magenta〉, |yellow〉, |green〉, are elements of some orthogonal basis. If we label the colors by numbers, we can write |0〉, |1〉, |2〉, |3〉, which suggests that |0〉=|cyan〉. But why cyan and not magenta or yellow? Can we make sense of ‘inequalities’ such as ‘cyan < magenta’? In fact, the order seems undefined by first principles, so formulas like

(137) $$\begin{array}{c} |\mathrm{cyan}\rangle =|0\rangle =|00\rangle =|0\rangle ⊗|0\rangle , \end{array}$$

are, to some extent, ambiguous. It turns out that this ambiguity is very interesting in itself, has status of a gauge freedom, and is linked to the old problem of completeness in quantum mechanics [28], or relations between correlations and correlata [29]. Further details can be found in [30].

## Data Availability

Data are contained within the article.

## Appendix A

### Appendix A.1. Multiple Products with Different Ns

The map (5)–(7) is one-to-one for any *N*. A composition of two such maps remains one-to-one even if each of them involves a different *N*,

(A1) $$\begin{array}{ccc} n & = & N_{0}k_{0}+l_{0}=N_{0}\left(N_{1}k_{1}+l_{1}\right)+l_{0}, \end{array}$$

where $0\leq l_{0}<N_{0}$, $0\leq l_{1}<N_{1}$. (A1) defines a bijection

(A2) $$\begin{array}{c} n\mapsto (k_{1},l_{1},l_{0}). \end{array}$$

Accordingly, by the same arguments as in Section 1, one is allowed to write

(A3) $$\begin{array}{ccc} |n\rangle & = & |N_{0}k_{0}+l_{0}\rangle =|k_{0},l_{0}\rangle \end{array}$$

(A4) $$\begin{array}{ccc} & = & |N_{0}\left(N_{1}k_{1}+l_{1}\right)+l_{0}\rangle =|k_{1},l_{1},l_{0}\rangle \end{array}$$

(A5) $$\begin{array}{ccc} & = & \left(|k_{1}\rangle {⊗}_{N_{1}}|l_{1}\rangle \right){⊗}_{N_{0}}|l_{0}\rangle =|k_{0}\rangle {⊗}_{N_{0}}|l_{0}\rangle , \end{array}$$

and so on, where we keep in mind the problem with associativity and nesting.

Orthonormality (3) is valid independently of the choice of $N_{i}$.

### Appendix A.2. Composition of Operators

Let

(A6) $$\begin{array}{ccc} A & = & \sum_{nm}|n\rangle A_{nm}\langle m| \end{array}$$

be some operator and we are interested in the relation between $\left(A{⊗}_{N_{1}}I_{N_{1}}\right){⊗}_{N_{0}}I_{N_{0}}$ and $A{⊗}_{N_{1}N_{0}}I_{N_{1}N_{0}}$.

(A7) $$\begin{array}{ccc} A{⊗}_{N_{1}}I_{N_{1}} & = & \sum_{k_{1}k_{1}^{\prime }l_{1}l_{1}^{\prime }}A_{k_{1}k_{1}^{\prime }}\delta_{l_{1}l_{1}^{\prime }}|k_{1},l_{1}\rangle \langle k_{1}^{\prime },l_{1}^{\prime }| \end{array}$$

(A8) $$\begin{array}{ccc} & = & \sum_{k_{1}k_{1}^{\prime }l_{1}l_{1}^{\prime }}A_{k_{1}k_{1}^{\prime }}\delta_{l_{1}l_{1}^{\prime }}|N_{1}k_{1}+l_{1}\rangle \langle N_{1}k_{1}^{\prime }+l_{1}^{\prime }| \end{array}$$

(A9) $$\begin{array}{ccc} \left(A{⊗}_{N_{1}}I_{N_{1}}\right){⊗}_{N_{0}}I_{N_{0}} & = & \sum_{k_{1}k_{1}^{\prime }l_{1}l_{1}^{\prime }l_{0}l_{0}^{\prime }}A_{k_{1}k_{1}^{\prime }}\delta_{l_{1}l_{1}^{\prime }}\delta_{l_{0}l_{0}^{\prime }}|k_{1},l_{1},l_{0}\rangle \langle k_{1}^{\prime },l_{1}^{\prime },l_{0}^{\prime }| \end{array}$$

(A10) $$\begin{array}{ccc} & = & \sum_{k_{1}k_{1}^{\prime }l_{1}l_{0}}A_{k_{1}k_{1}^{\prime }}|k_{1},l_{1},l_{0}\rangle \langle k_{1}^{\prime },l_{1},l_{0}| \end{array}$$

(A11) $$\begin{array}{ccc} & = & \sum_{k_{1}k_{1}^{\prime }l_{1}l_{0}}A_{k_{1}k_{1}^{\prime }}|N_{0}N_{1}k_{1}+N_{0}l_{1}+l_{0}\rangle \langle N_{0}N_{1}k_{1}^{\prime }+N_{0}l_{1}+l_{0}| \end{array}$$

Variable $l_{01}=N_{0}l_{1}+l_{0}$ satisfies

(A12) $$\begin{array}{c} 0\leq l_{01}\leq N_{0}N_{1}-1 \end{array}$$

Proof:

(A13) $$\begin{array}{ccc} 0\leq N_{0}l_{1}+l_{0} & \leq & N_{0}\left(N_{1}-1\right)+N_{0}-1 \end{array}$$

(A14) $$\begin{array}{ccc} & = & N_{0}N_{1}-N_{0}+N_{0}-1=N_{0}N_{1}-1 \end{array}$$

Thus,

(A15) $$\begin{array}{ccc} \left(A{⊗}_{N_{1}}I_{N_{1}}\right){⊗}_{N_{0}}I_{N_{0}} & = & \sum_{k_{1}k_{1}^{\prime }}\sum_{l_{01}=0}^{N_{0}N_{1}-1}A_{k_{1}k_{1}^{\prime }}|N_{0}N_{1}k_{1}+l_{01}\rangle \langle N_{0}N_{1}k_{1}^{\prime }+l_{01}| \end{array}$$

(A16) $$\begin{array}{ccc} & = & \sum_{k_{1}k_{1}^{\prime }}\sum_{l_{01}=0}^{N_{0}N_{1}-1}A_{k_{1}k_{1}^{\prime }}\left(|k_{1}\rangle {⊗}_{N_{0}N_{1}}|l_{01}\rangle \right)\left(\langle k_{1}^{\prime }|{⊗}_{N_{0}N_{1}}\langle l_{01}|\right) \end{array}$$

(A17) $$\begin{array}{ccc} & = & A{⊗}_{N_{1}N_{0}}I_{N_{1}N_{0}} \end{array}$$
